# Supplementary material for: A Randomized Cross-Over Study Comparing Cooling Methods for Exercise-Induced Hyperthermia in Working Dogs in Training
Source: Animals (Basel). 2023 Nov 28;13(23):3673. doi: 10.3390/ani13233673 (PMC10705156; doi:10.3390/ani13233673)
Supplement: Supplementary file 1 [file animals-13-03673-s001.zip › animals-2705475-supplementary.pdf]

Supplementary Tables

Table S1. Study Participant information
This table contains descriptive information about the canine participants in the study. Age is presented in months and weight is presented in pounds. Body condition score is on a 9-point scale and was evaluated by a veterinarian. Dogs were randomly assigned to a group at the start of the study.

Participant Number	Breed	Age (months)	Sex	Weight in pounds (mean of trial days)	Body Condition Score	Group Number
1	Dutch Shepherd	11	Female	47.4	4.0	1
2	German Shepherd	80	Female	73.1	5.5	1
3	Labrador Retriever	13	Male	68.4	5.0	1
4	Labrador Retriever	63	Female	49.2	4.5	2
5	German Shepherd	101	Female	64.3	5.0	2
6	Labrador Retriever	10	Female	50.6	5.0	2
7	Labrador Retriever	13	Female	53.7	5.0	2
8	Dutch Shepherd	14	Female	46.4	4.0	2
9	German Shepherd	13	Male	76.9	6.0	1
10	Labrador Retriever	20	Female	51.7	4.5	1
11	Labrador Retriever	20	Female	63.4	4.5	2
12	Labrador Retriever	16	Male	67.9	6.5	1

Table S2. Trial day weather information
This table shows the average temperature, humidity, and heat index on each of the study days, as well as the standard deviation. Temperature and humidity are presented in Fahrenheit and Celsius. Humidity is presented as a percentage.

Date	Average Ambient Temperature: ºF (ºC)	Standard Deviation of Ambient Temperature: ºF (ºC)	Average Humidity (%)	Standard Deviation of Humidity (%)	Average Heat Index: ºF (ºC)	Standard Deviation of Average Heat Index ºF (ºC)

7/18/22	83.2 (28.4)	1.7 (1.0)	76.7	4.5	90.5 (32.5)	3 (1.7)
7/19/22	80.2 (26.8)	1.5 (0.8)	64.5	4.3	83.8 (28.8)	1.6 (0.9)
7/21/22	83.8 (28.8)	0.84 (0.5)	75.2	1.8	91.8 (33.2)	1.3 (0.7)
7/22/22	83.7 (28.7)	1.4 (0.8)	55.0	3.6	87.0 (30.6)	0.9 (0.5)
7/26/22	74.0 (23.3)	0.7 (0.4)	57.8	2.5	74.0 (23.3)	0.7 (0.4)
7/29/22	75.6 (24.2)	0.5 (0.3)	79.8	1.1	75.6 (24.2)	0.5 (0.3)
